# Inter-rater reliability data of classroom observation: Fidelity in large-scale randomized research in education

**DOI:** 10.1016/j.dib.2020.105303

**Published:** 2020-02-17

**Authors:** Fuhui Tong, Shifang Tang, Beverly J. Irby, Rafael Lara-Alecio, Cindy Guerrero

**Affiliations:** a Center for Research and Development in Dual Language and Literacy Acquisition, Department of Educational Psychology, Texas A&M University, College Station, TX 77843, United States; b Education Leadership Research Center, Educational Administration and Human Resource Development, Texas A&M University, College Station, TX 77843, United States

**Keywords:** Inter-rater reliability, Classroom observation, Fidelity of implementation, Bilingual/ESL education

## Abstract

This dataset belongs to a large-scale randomized controlled trial (RCT) in educational research targeting English learning students and their teachers' instructional capacity. The dataset includes ratings conducted through classroom observations of 45-minute English as a Second language (ESL) blocks. Each coder rated 60 recorded video segments collected from each teacher. During the 20-second segment, ratings of six domains of teachers' instruction (i.e., ESL Strategies, Group, Activity Structure, Mode, Language Content, Language of Teacher, Language of Student) were collected. The dataset is organized by teacher, by coder, and by domain, for researchers to analyze inter-rater reliability among coders by domain and/or cross-domain. This data article is related to the research article Tong et al. [3] on “The determination of appropriate coefficient indices for inter-rater reliability: using classroom observation instruments as fidelity measures in large-scale randomized research”.

**Table:** Specifications Table

Subject	Social Sciences
Specific subject area	Education; bilingual/English as second language (ESL) education
Type of data	Table
How data were acquired	Virtually-recorded classroom videos were coded via an online coding platform http://tbop.teachbilingual.com/
Data format	Cleaned and labelled raw data
Parameters for data collection	Recordings were collected during a 45-minute block of ESL instruction. Research staff and graduate students of the project rated the recordings using a multi-dimension-multi-response (MDMR) instrument, i.e., Transitional bilingual observation protocol (TBOP, [1,2]). The coding was based on the following dimensions: ESL Strategies, Group, Activity Structure, Mode, Language Content, Language of Teacher, and Language of Student. These coders received training provided by the research team who were the developers of the observation instrument.
Description of data collection	Classroom instruction was recorded virtually. Videos were then coded by trained personnel. A value representing the category in each dimension is recorded to indicate the presence of a certain pedagogical behaviour.
Data source location	College Station, Texas, the United States
Data accessibility	With the article or Mendeley Data link https://data.mendeley.com/datasets/479hwxdfwb/draft?a=10b48a38-dc39-46b3-8402-4ffbe51548f3
Related research article	Tong, F., Tang, S., Irby, B. J., Lara-Alecio, R., & Guerrero, C., (2020). The Determination of Appropriate Coefficient Indices for Inter-Rater Reliability: Using Classroom Observation Instruments as Fidelity Measures in Large-Scale Randomized Research. International Journal of Educational Research [3].

**Table:** 

**Value of the Data** • Classroom observation is recommended as an objective approach to measuring fidelity of implementation (FOI) in experimental research [[4], [5], [6]]. The establishment of inter-rater reliability of observation instruments is receiving more attention [7,8]. Our FOI data were acquired via classroom observations with extensive resources and personnel and quality training in the most rigorous design in educational research through a U.S federal-funded project. • The empirical dataset presents multiple coders' rating of an MDMR protocol with nominal data (i.e., TBOP) that were used for FOI in an RCT on improving elementary bilingual teachers' instructional capacity and their ELs' language achievement in English. • The dataset can be used to conduct inter-rater analyses and calculate different indices of inter-rater agreement. It can be used to compare different agreement indices in order to test the levels of reliability related to MDMR data in the bilingual/ESL context. • The dataset demonstrates a scientific approach to record data for rater agreement when there are multiple raters involved in classroom observation, which is informative to practitioners, researchers, and administrators in their future data analysis in educational research where public data are not readily available regarding classroom observation.

## 1 Data description

The dataset contains seven observation dimensions: ESL Strategies, Group, Activity Structure, Mode, Language Content, Language of Instruction/Teacher, and Language of Student that were compiled in one workbook (see Table 1). Each sheet (tab) was named after the domain of observation. The top row contains variable names: teacher (1–3), segment (1–60), and coder (1–6). The first column is the index of three teachers whose lessons were recorded and coded. The second column is the index of segments that were coded. Each video is coded into 60 segments, and each segment lasts 20 seconds. Columns 3–8 are coders' ratings of each segment. For example, column 3 is Coder 1's ratings of three videos of 60 20-second segments that were rated. The value in each cell from columns 3–8 represents a unique code corresponding to an instructional activity within each domain that was observed by the coders during that segment. The explanation of these codes and coding protocol can be referred to Lara et al. [1] and Tong et al. [2]. These data are therefore nominal in nature and can be used to calculate different indices of inter-rater agreement as was reported in Tong et al. For future analysis, researchers need to select appropriate inter-rater indices for the nominal data in educational research.

**Table 1:** Descriptive statistics of the data used to calculate inter-rater reliability.

# of teachers	# of domains (worksheets)	# of coded 20-second segments	# of coders
3	7	180	6

## 2 Experimental design, materials, and methods

Data in this paper were part of a larger database of classroom observation from a randomized project. We randomly selected 10% of the data to ensure sample representativeness. The purpose of obtaining such a sample was to calculate interrater reliability among the coders of their observations. We recommended 10% in consideration of the total number of raters involved in this process (between 5 and 8, see Tong et al. [2]). After receiving intensive training of the observation instruments, each coder was assigned three recorded lessons to code individually with the intent to reach inter-rater reliability. In the coding process, the coders watched the first five-minute of the recorded video to obtain a general sense of the lesson. In the next five minutes, the rater coded 15 segments with each segment lasting for 20 seconds. The coders repeated such circle four times till he/she completed coding 60 20-second segments of a 45-minute ESL lesson. For each 20-second segment, a coder rated the above-mentioned six domains. The data presented in the paper were cleaned and organized by domain in the order of 20-second segments that were coded for each teacher.
